# A Survey Exploring How Watch Officers Manage Effects of Sleep Restrictions during Maritime Navigation

**DOI:** 10.3390/ijerph20020986

**Published:** 2023-01-05

**Authors:** Claire Giot, Laure Lejeune, Nicolas Bessot, Damien Davenne

**Affiliations:** 1 UNICAEN, INSERM, COMETE, Normandie Université, 14000 Caen, France; 2 UNICAEN, ENSICAEN, CNRS, GREYC, Normandie Université, 14000 Caen, France

**Keywords:** maritime officers, shift work, sleep deprivation, sleepiness, sleepiness countermeasures

## Abstract

Merchant marine officers work shifted hours with a sometimes very tiring work/rest rhythm that can lead to sleep restrictions and increased sleepiness during navigation. The aim of this study is to assess the risk of sleep deprivation-related sleepiness during navigation and the factors contributing to this risk. A second objective is to evaluate the use and effectiveness of sleepiness countermeasures. An online quantitative survey of 43 questions was conducted on 183 French maritime officers. A total of 39.9% of the participants experienced at least occasionally severe sleepiness and 29% had fallen asleep during navigation. A total of 42.6% reported not being able to experience enough sleep on board. Sleep requirements were affected by time spent on board, area of activity, and watch system. Sleepiness was more common during monotonous than demanding sailing. Officers frequently use caffeine, as well as vigilance-enhancing activities that they consider effective, which are not yet validated, (i.e., social interactions). However, they are not inclined to seek replacements in case of severe sleepiness. Sleep deprivation is common among maritime officers and leads to the risk of severe sleepiness while operating the vessel, with few effective countermeasures available. Strategies used for sleep management and sleepiness prevention should focus more on sleep duration, safety culture, and improving countermeasures to sleepiness.

## 1. Introduction

Merchant marine officers work both day and night, so they often have staggered hours and, in some cases, a very tiring work/rest rhythm [1]. Shift work is associated with an increased risk of accidents in the transport and industrial sectors [2,3]. One of the main explanations is a reduction of sleep times and an activity sometimes performed at night, during the batyphase of the circadian cycle of alertness [4,5]. In addition to these temporal constraints, watch officers’ sleep is likely to be interrupted by ship noise and motion [6]. Indeed, in the scheduled hours of watchkeeping for navigation, officers have multiple duties and may be required to work a number of hours exceeding what is required by law, further impacting their rest and sleep period. Attempts by the International Maritime Organization (IMO) to regulate have so far been ineffective, as seafarers themselves are prone to falsifying reports of the number of hours worked [7,8]. In addition to their influence on officers’ health, these sleep conditions may induce a risk of loss of attentional capacity impacting navigation safety. Lack of sleep due to acute or chronic sleep deprivation is the main cause of sleepiness [9] which is a major cause of vigilance decrement. Maritime officers are responsible for managing ship navigation and must keep a constant and satisfactory watch on the maritime environment [10]. The officer must also be able to apply steering rules, monitor the condition of the ship, and be able to react to damage or risk of collision. It appears that a good level of vigilance is necessary to conduct a vessel and detect changes in the environment. For maritime officers, sleep restrictions can lead to severe sleepiness and even falling asleep on deck, especially at night [11]. One in four officers admitted to falling asleep on watch [7]. The activity of steering a ship is highly automated and does not require constant action from the officer, as does driving a car, which explains that sleepiness or falling asleep may not always have immediate consequences. However, these events are frequently found in accident reports, where it is stated that the officer fell asleep or lacked vigilance, missed observations, or overlooked the upcoming seabed [12].

Investigations carried out on merchant navy officers mainly focused on the impact of watch schedules on sleep. The steering of a ship must be carried out without interruption 24 h a day and to meet this requirement, ship officers usually work split shifts. This system is characterized by the division of a 24-h day into successive work and rest periods. In the merchant marines, schedules are generally fixed and the “4-on/8-off” system is the most widespread. Three teams work 24 h, each doing two 4-h shifts, one during the day and the other at night, separated by 8 h of rest [13]. Other watch systems may also be found, such as the ‘6-on/6-off’ system, as is the case on coastal vessels, where two officers are assigned to the bridge, alternating periods of 6 h on and 6 h off over 24 h. A distinction can be made between officers who practice different watch systems. The 6-on/6-off system was associated with more excessive sleepiness [11] and more accidents than the 4-on/8-off system [14] due to shorter sleep and recovery time. With regard to work organization, it can also be noted that the interruption of free watch periods, common during passages with frequent port calls, will prevent officers from resting and napping and greatly increase the level of sleepiness [15]. In a 4-h shift system with 8 h off, subjective (KSS) and objective (PVT) sleepiness peaks happened during night and early morning shifts, which coincides with the period when a relatively high number of maritime accidents occur [11,15]. Sleepiness is generally low at the beginning of the shift [16], and peaks at the end where longer reaction times are observed [15]. As the duration of the task increases, severe sleepiness increases and becomes greater for longer shifts [17]. The impact of organizational or activity-related factors on sleep opportunities and sleepiness during the watch would, however, need to be further explored.

In order to address the effects of work patterns on health and safety, various countermeasures have been implemented. A distinction can be made between proactive measures that prevent the onset of fatigue by acting on sleep and sleep hygiene (adequate sleep duration, quality of sleep…) and reactive measures (or self-administered countermeasures) that act on fatigue at the time it occurs [18]. The International Maritime Organization [19,20] has issued a series of recommendations to encourage companies to manage fatigue and to facilitate rest periods and sleep hygiene on board. The question that is currently being asked is whether these policies have an effect on sleep opportunities and sleepiness during navigation. Another option to counteract the effects of sleep deprivation is to use countermeasures that can temporally enhance the level of arousal whilst not compensating for the sleep debt. Therefore, IMO also recommends that officers apply a set of self-administered countermeasures: short rest breaks within duty periods; caffeine; physical activity; social interaction; a stimulating environment (cool dry air, music, and other irregular sounds); changes in work routine and controlled naps (20 min). Countermeasures such as napping or intake of caffeine have shown their effectiveness to restore alertness in other contexts, such as driving [21,22], while the effect of others, such as social interaction, remain uninvestigated. Although they are recommended, the use and efficiency of these measures have not been systematically evaluated in the maritime context [23], and during maritime navigation. This might be due to the difficulty of accessing the population and evaluating them in context. Within maritime officers, only one study addressed this issue and indicated a potential effect of napping on sleep debt accumulation in marine pilots [24]. Thus, it seems relevant to evaluate the use of countermeasures and possibilities of remediation when the officer feels too drowsy to continue sailing. A questionnaire could be an accessible way to reach maritime officers.

It appears that the work rhythms and conditions on board are likely to strongly affect the sleep of merchant marine officers with a risk of sleepiness or even falling asleep while operating the ship. Recommendations exist concerning sleep hygiene and countermeasures that can be applied by officers to counteract sleepiness, as well as on the behavior to adopt in case of significant sleepiness. Their actual use and their effectiveness must however be further explored.

The first objective is to evaluate the risks of mild to severe sleepiness during the watch related to sleep deprivation on board, as well as the factors favoring these risks among merchant marine officers. These results will also allow us to update existing data on sleepiness among officers.

Secondly, we evaluate the use of countermeasures during maritime navigation and the possibilities of remediation in the officers, as well as their perceived effectiveness.

## 2. Materials and Methods

### 2.1. Procedure

The study was conducted among French maritime officers between March and June 2022. It consisted of a self-administered questionnaire to be completed online, which was addressed to all bridge watchkeeping officers regardless of their level of experience. The questionnaire included demographic as well as job-related characteristics, sleepiness experience, countermeasures use, and perceived-efficiency (see Section 2.3 “questionnaire content” for a comprehensive presentation of the questionnaire items). Prior to the final distribution of the questionnaire, a pilot test was conducted with three officers of the watch with different levels of experience and ranks (Master, Chief officer and Mate). Preliminary cognitive interviews were conducted to eliminate points of misunderstanding (ensure the relevance of the terms used with the maritime context and a good understanding of the questions’ goal) and any gaps between the survey’s intentions and the way respondents understand questions. Respondents were asked to complete the questionnaire and comment on it. They were also asked additional questions about how the questions were understood. This resulted in the adaptation of some of the formulations and in the inclusion of additional response options. 

Then, the Lime survey platform was used to construct the online questionnaire and to collect the responses. The questionnaire link was disseminated via a journal aimed specifically at merchant marine officers as well as via the shipmasters of French shipping companies for merchant marine officers. The data have been collected anonymously in compliance with the General Data Protection Regulation EU-2016/679. The data collected and their processing were investigated by Caen University’s Data Protection Officer. Information notice was given to participants and informed consent was collected.

### 2.2. Population

Two hundred and four people answered the questionnaire, and twenty-one participants were removed from the sample as they did not meet the research criteria (no experience at sea, not working on a commercial ship for several years, or not being assigned to watchkeeping). A total sample of 183 merchant navy officers was considered in the study (172 males and 11 females), with ages ranged from 21 to 67 years, and an average age of 34.6 years (SD = 8.8 years). All participated in the navigation of the ship, either as ship’s Master (30.1%), Chief officer (40.4%), or Mate (29,5%). Participants in our sample were experienced seafarers, with an average navigation experience of 9.89 years (SD = 8.2). At the time of the survey, all the participants reported sailing for at least 6 months or more. Among them, 36.6% were regulated under the French flag state, 50.8% under French International Register (RIF), and 12.6% under another flag state. The main sectors involved were passenger transport (33.9%), freight transport (24%), and specialized vessels such as coastal or offshore vessels (26.2%). The other sectors (tugs, service vessels, etc.) represented 15.8% of the sample. All participants were required to work at night, 88% on a very regular basis (i.e., several times a week or every day).

### 2.3. Questionnaire Content

In order to meet the objectives of the study, the questionnaire collected data concerning participants’ demographics and working conditions, sleepiness experience, and the strategies they use to counteract sleepiness as well as their perceived efficiency.

#### 2.3.1. Demographics and Working Conditions

The questionnaire considered variables related to the respondents’ individual characteristics and working conditions in order to assess their effect on sleepiness while operating a vessel.

Shift-work organization was evaluated as it is the most likely to influence sleepiness and the amount of sleep. The duration of the trips at sea and the Maritime sectors (e.g.,: passenger transport, freight, etc.) were also questioned as they may impact work rhythm and workload. As it is a main cause of sleepiness, participants were also asked if they had the possibility to sleep enough on the boat to meet their sleep needs. The sleep duration has not been exactly evaluated as this is likely to vary significantly depending on the activity and over the days on board.

#### 2.3.2. Sleepiness Experience

The following questions were about their experience of sleepiness while on watch on the bridge. Participants had to answer according to their experience over the past two years. A sleepiness definition was provided to the participant. It has been defined as: “a desire to sleep that can occur at any time of the day. It is a momentary decrease in wakefulness with difficulty in concentrating” In order to determine the conditions contributing to the occurrence of sleepiness, a 5-point Likert scale (from “never” to “very often”) evaluated the frequency of occurrence of drowsiness during the day and the night, as well as in relation to specific activity (calm and monotonous navigation conditions, heavy traffic, and tight maneuvers). A somnolence score was built based on six sub-questions from the Sleepiness Symptoms Questionnaire [25] which rated the frequency of symptoms of sleepiness on a scale from “not at all”, “rarely “, “occasionally”, “frequently” and “most of the time”. (“Struggling to keep your eyes open”, “vision becoming blurred”, “nodding off to sleep”, “mind wandering to other things”, “reaction slow”, “head dropping down”). SSQ items that were related to driving were removed as non-relevant (“Difficulty keeping to middle of the road” and “Difficulty maintaining correct speed”). Finally, we asked participants the frequency with which they suffer from severe sleepiness (“I struggle to stay awake”) and if they had already fallen asleep on a bridge watch.

#### 2.3.3. Use and Perceived-Efficiency of Countermeasures, Other Possibilities of Remediation

We evaluated the frequency of use of countermeasures considered effective in restoring vigilance or recommended by the IMO to seafarers on a 5-point Likert scale (from “never” to “very often”): short rest breaks within duty periods, caffeine, physical activity (moving around, walking, or stretching), social interaction (talking with colleagues), cool air, music, or podcast listening; changes in work routine and naps. The practice of controlled anticipated naps before the shift was also questioned. Plus, we assessed the other possibilities of remediation, namely the frequency of requesting a replacement for excessive drowsiness during the shift and the request for a replacement for inaptitude on duty, and asked participants how often they continued to navigate while feeling too tired to do so with a 5-point Likert scale. Last, participants had the possibility to rate the efficiency of each countermeasure on sleepiness with a 4-point Likert scale («not effective»; «rather not effective»; «rather effective»; «very effective») or they also had the possibility to answer that they did not know.

### 2.4. Statistical Analysis

SPSS Statistics 26 software was used for data analysis. Non-parametric statistics were used to test the effect of work-related variables and countermeasures on the Likert scales. The Mann-Whitney U test for independent samples was used for variables with two independent groups and the Kruskal Wallis test if there were more than two groups. Chi-Square test was used to test the association between categorical variables. Friedman’s two-way ANOVA was used to compare the sleepiness scores between the different navigation conditions. When the conditions for parametric statistics were met, a student’s t-test was used to compare the scores on the numerical variables.

Only the three main sectors (passenger transport (33.9%), freight transport (24%), and specialized vessels such as coastal or offshore vessels (26.2%) were considered for the “sector” variable, as other sectors were very scarcely represented among the respondents. For the “duration at sea”, the sample was split at the median, between participants who embarked for less than two months and those who embarked for two months. Two groups of participants were also created based on their number of experienced years on watch. The cut-off was made at the median of the sample with an “experienced” group above 8 years of practice and a “short experience” group below 8 years. The difference between shift durations was calculated for the 4 on-8 off; 6 on-6 off and 12 on-12 off shift systems, as the other shift systems were very infrequent in the answers and uncommon in the population of watch officers.

## 3. Results

### 3.1. Sleep Requirement and Frequency of Mild to Severe Sleepiness during Navigation

In our sample, 42.6% of officers surveyed reported not be able to experience enough sleep to fulfil their sleep needs. In the past two years, 39.9% experienced severe sleepiness during navigation (struggling to stay awake) at least occasionally, including 10.9% frequently or most of the time and 29% of the participants having already fallen asleep once during watch duty.

### 3.2. Effect of Individual Factors and Work Organization on Sleepiness and Sleep Fulfilment

A composite sleepiness score was built from the average of the responses to the six items from the SSQ (m = 2.21; sd = 0.67), the value for Cronbach’s Alpha was α = 0.83, corresponding in good internal consistency.

The effect of individual factors (age, function, experience), factors related to working conditions (maritime sector), and work rhythm (the duration of the trips at sea, the shift system, and the possibility of experiencing enough sleep to meet sleep needs) were tested on sleepiness score and severe sleepiness. The possibility of experiencing enough sleep to meet sleep needs was the only factor that was significantly related to sleepiness score based on SSQ (t (181) = −4.394, *p*= 0.000) and to severe sleepiness frequency (U = 1843, *p* = 0.000).

Further analyses were conducted to find out which factors influenced the possibility of experiencing enough sleep on board. Sleep requirements were more likely to be fulfilled by participants who sailed for two months or more rather than those who navigated for a shorter duration (χ^2^(1) = 9.376, *p* = 0.002). The sector of activity also has an impact on the possibility of sleeping (χ^2^(2) = 10.799, *p* = 0.005), especially in passengers transport, where 65.5% of the officers were unable to sleep enough on board compared to 34.1% in specialized ships and 41% in freight transport. The shift system also had an effect on sleep opportunity (χ^2^(2) = 6.827, *p*= 0.033), with the 12 on-12 off system appearing to be the most beneficial, with 78.3% of participants reporting experiencing sufficient sleep, compared to 51% for the 4 on-8 off system and only 43.5% for the 6 on-6 off system. The distribution of the responses for each factor is presented in Table 1.

### 3.3. Effect of Shipping Conditions on Sleepiness

**Figure 1:**
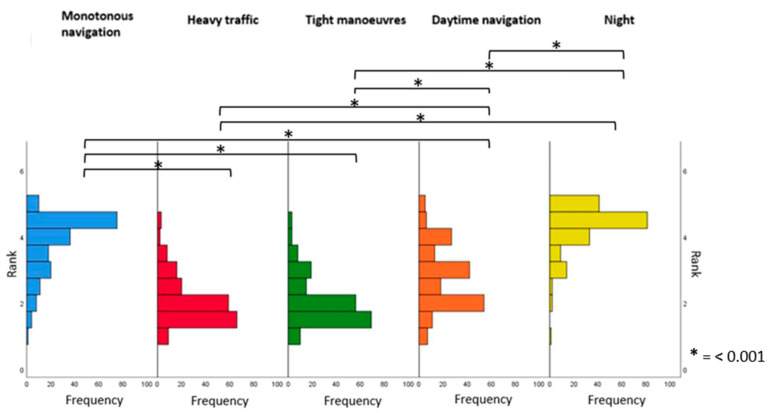
Frequency of sleepiness as a function of navigation condition.

Finally, we tested the effect of shipping conditions on sleepiness frequency. A Friedman test followed by post-hoc tests were carried out and adjusted using the Bonferroni correction to examine the effect of navigation contexts on sleepiness frequency. Results showed that the context of navigation leads to statistically significant differences in sleepiness occurrence (Q = 420.891, *p* < 0.001). As expected, sleepiness was more frequent at night than during the day (*p* < 0.001). Sleepiness was less frequent during periods of activity (traffic or tight passage) than in other conditions (*p* < 0.001). Sleepiness during monotonous passages had a frequency that was not significantly different from that experienced at night (*p* = 0.082). Results are presented in Figure 1.

### 3.4. Use and Perceived-Efficiency of Sleepiness Countermeasures during the Watch

**Figure 2:**
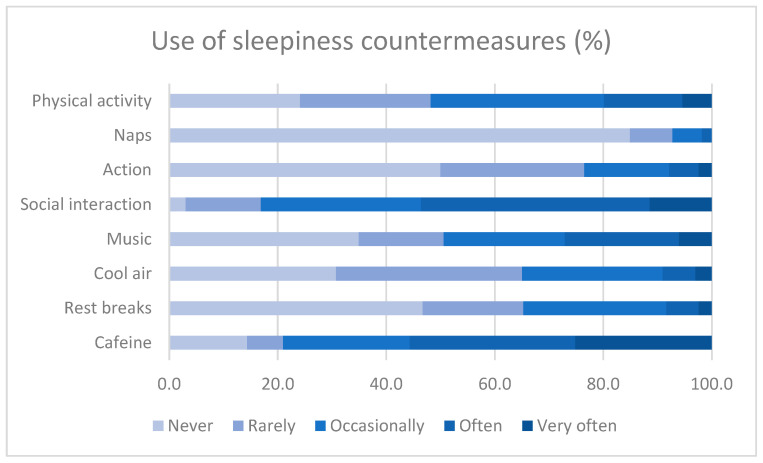
Frequency distribution for the use of countermeasures during navigation.

If we compare the measures on the reported regular frequency of use (“often” and “very often”) with each other, caffeine appears to be the most regularly used countermeasure in our sample (55.7% use it often or very often), followed by social interaction (53.6%), listening to music (27.1%), and physical activity (19.9%), while the other countermeasures (naps, action) are each used regularly by less than 10% of the respondents. Figure 2 illustrates the frequencies of use for each countermeasure. In order to prevent drowsiness during the watch, 80.1% of the officers also take preventive naps outside the navigation periods.

**Figure 3:**
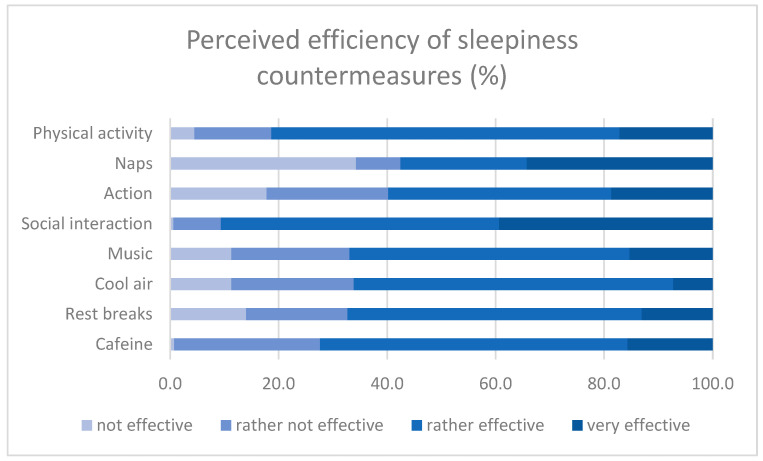
Frequency distribution for the perceived efficiency of sleepiness countermeasures.

Social interaction is considered an efficient countermeasure (classified as “rather effective” or “very effective”) by 90.6% of the respondents, followed by physical activity (81.3%), caffeine (72.4%), rest breaks (67.3%), music (66.9%) cool air (66.1%), and naps (57.5%). It is noteworthy that napping is considered a “very effective” way of counteracting sleepiness by 34.2% of respondents, second to social interaction which has the largest proportion of “very effective” ratings (39.4%). Figure 3 depicts the complete distribution of answers.

### 3.5. Other Strategies in Case of Severe Sleepiness

Only 9% of the respondents have ever asked not to take a shift due to severe fatigue. When they feel sleepy on the bridge, 76.5% do not ask to be replaced, 15.3% do so rarely, 5.5% occasionally, and less than 0.5% do so often. Finally, 36.6% of participants say they sail while feeling too tired to continue at least occasionally (including 7.1% often and 1.9% very often).

The position held on board has a significant effect on asking to be replaced in case of severe sleepiness. A Kruskal-Wallis H test showed that there was a statistically significant difference in replacement frequency between the different categories of officer (χ2(2) = 14,691, *p* = 0. 001). Pairwise tests were carried out for the three pairs groups and adjusted using Bonferroni correction. Masters are more likely to ask to be replaced (12%) than Lieutenants (6.7%, *p* = 0.001) or Chief officers (4.8%, *p* = 0.003).

## 4. Discussion

This study was conducted to evaluate the risks of sleepiness during the watch related to sleep deprivation on board, as well as the factors favoring these risks among merchant marine officers. A second objective was to evaluate the use of countermeasures during maritime navigation as well as their perceived effectiveness and the other possibilities of remediation existing for officers. Results highlighted that the frequency of severe sleepiness during navigation should be a matter of concern, sometimes leading to falling asleep for almost a third of officers. The only factor influencing the frequency of sleepiness and severe sleepiness was found to be the inability of the officer to experience enough sleep on board to meet his sleep requirements.

The possibility of experiencing enough sleep was affected by factors linked to work organization and rhythms: duration on board, sector of activity, and watch system. In addition, the conditions in which the watch was kept had an impact, with navigation in calm, monotonous traffic being associated with more frequent sleepiness. The two countermeasures that participants most often reported using regularly were caffeine and social interactions. They were also the most frequently reported as being efficient countermeasures with physical activity. The results also indicate that officers rarely call for a replacement in case of severe fatigue during the shift, and that few have ever given up taking a shift due to severe fatigue.

### 4.1. Factors Contributing to Sleep Deprivation and Drowsiness during Navigation

The results concerning the frequency of severe sleepiness and falling asleep are consistent with previous studies [7,11,15]. They highlight the severity and continuing importance of these risks in the merchant marines. Our findings indicate that the possibility of experiencing enough sleep is affected by factors linked to work organization and rhythms: boarding time, sector of activity, and watch system. The first two factors are linked to the organization of work and can have an impact on rest periods. Longer embarkations are generally associated with longer time in the open sea that will allow seafarers to recuperate more efficiently [26], while shorter embarkations and passenger transport are associated with more frequent stopovers, leading to an increase in workload outside of shifts [27]. The results also indicate that while the length of the shift may have an impact on sleep duration, the prevalence of sleepiness is not significantly different between the different shift systems, and half of the 4 on-8 off workers report not experiencing enough sleep. There is, therefore, a possible discrepancy between the scheduled rest opportunities and what is actually possible due to the actual workload. On this point, the improvement of sleep is strongly dependent on legislation and organizations (policy of management by the company and team management), as it requires a work organization that is adjusted to respect proper work/rest scheduling. Concerning shift systems, there might be a need for longer “off” periods to account for the activities surrounding sleep, the time to fall asleep, and sleep inertia. Some new shift systems have been designed to provide longer rest periods, as for example the “Swedish shift” which proposes to have a 3-h and then 5-h watch periods during the day, allowing 10 continuous hours of rest. Another issue, however, is maintaining synchronization with the 24-h circadian rhythms. A study of 145 seafarers compared reaction times, a parameter that is sensitive to sleepiness, across four different types of schedules (3/9, 6/6, 6/18, 5/10) [28]. Staff in 5-on/10-off were the most out of sync and performed the worst on the reaction time test (PVT) followed by staff in 6-on/6-off. The best performance was by staff in 3-on/9-off. While improving sleep duration is a crucial prerequisite, the effect of the disruption to the circadian rhythm that occurs with shift work and night work, will nonetheless impact sleep and induce sleepiness [29]. In addition, sleep duration and quality on board also depend on the infrastructure (comfort and sound insulation) [30] and on the weather conditions. Thus, the state of alertness of the officers operating the vessel is impacted by a combination of factors that must all be considered in order to reduce the risks.

### 4.2. Use of Countermeasures to Sleepiness during the Watch

This study enabled the quantification of the use of countermeasures to sleepiness during maritime navigation. The use of countermeasures to the sleepiness has been evaluated in the road sector [31] but not comprehensively in maritime navigation although they are recommended as strategies to reduce sleepiness. First, the use is influenced by what is possible on board and what is allowed. In practice, there are more possibilities than in a car or a truck, for example, because the officer can move around the bridge, have coffee, etc. Caffeine is regularly used as a countermeasure as well as social interactions that are easy when there are several people on the navigation bridge. The use of other countermeasures is limited by navigation activity and regulations. For example, “active” piloting is not always possible or necessary in a ship, and in fact, cannot be used frequently. Physical activity will be limited to taking a few steps or stretching and is possible when the navigation activity is not too demanding, as is the case for rest breaks, while music is not always allowed. The short nap is rarely used during the watch because it is forbidden, even when the officer is not alone on the bridge, whereas a large majority of officers take preventive naps outside of navigation periods to avoid the occurrence of sleepiness.

### 4.3. Perceived-Efficiency of Countermeasures to Sleepiness during the Watch

Based on the work of Anund [32] and Pylkkönen [33], countermeasures can be categorized into different clusters of behaviors: supplementary rest breaks (breaks other than statutory), napping and caffeine ingestion, and alertness-enhancing activity. Regarding the countermeasures evaluated in this study, coffee and napping are the only ones validated as efficient to restore alertness [34]. In our results, coffee is judged overall to be rather effective by the majority of participants. The more contrasting results for napping while a third of the participants consider it “very effective”, can be explained by the fact that napping is prohibited and might be considered undesirable during navigation. It is also possible that naps during navigation are too short to have an effect or may be involuntary consequences of excessive sleepiness when it occurs. In this questionnaire, we evaluated the effectiveness of napping only as a countermeasure used during navigation, which may contribute to this result. Although the other countermeasures evaluated are not validated to counteract sleepiness [33], they were considered rather effective by the majority of participants. Indeed, they may have at least an indirect effect by enhancing alertness and counteracting factors inducing sleepiness other that sleep deprivation [35]. The fact that socializing is considered very effective makes sense when considering the impact of monotonous navigation on sleepiness. This may be considered effective in reducing the monotony, although it does not counteract the effect of sleep deprivation on physiology. The same cause is relevant for action (judged effective but less used because of limited possibilities). In addition to stimulation, socializing means not being alone in the gateway and having support for the watch when fatigued, which can reinforce the perception of efficiency. Physical activity (movement, stretching) is also often judged very efficient. While the activity possible during navigation may be a short and irregular effort in contrast to what was tested in research [36], it will still provide stimulation and in the context of maritime navigation, will prevent falling asleep by not sitting still. However, despite their perceived effectiveness, the actual effect of these measures in reducing sleepiness should be evaluated, as none of them, apart from caffeine, has been validated. Furthermore, the officer’s options for counteracting sleepiness while navigating remain limited. The high rate of severe drowsiness and falling asleep during the watch reported in this study, indicate that it is also necessary to test and develop effective measures applicable directly to the navigation bridge. Currently, there is also an electronic system that aims to prevent the officer from falling asleep or fainting during the watch. This system, named BNWAS (Bridge Navigational Watch and Alarm System), will trigger an alarm at regular intervals, approximately every 12 min, that the officer must turn off by pressing a control, or which is deactivated by a motion detector. Unfortunately, it appears that this system, which is considered to be cumbersome, is frequently deactivated by the officers.

### 4.4. Other Strategies of Remediation: Replacement in Case of Severe Sleepiness

Another issue is related to the behavior that should be taken when the officer is able to identify his or her sleepiness and considers himself or herself to be unfit for duty. The results indicate that despite the prevalence of severe drowsiness, very few officers have ever withdrawn from a shift due to severe fatigue, and that they are reluctant to be replaced if they feel the need to do so, with Masters making slightly more use of it. The difficulty of being replaced may be understandable insofar as it implies the involvement of another officer and therefore impacts on colleagues, but these results are worrying as they indicate difficulties in asking for help if necessary and possibly a lack of safety culture. Furthermore, it questions the application and effectiveness of the regulations which require the officers to be fit for duty when they are responsible for the watch.

### 4.5. Strengths and Limitations

The questionnaire is a means of reaching a population that is difficult to access for direct measurement and obtaining a large number of responses. However, it provides a subjective measure of sleepiness. A limitation is the difficulty of self-assessing and correctly estimating one’s level of sleepiness [37]. For this reason, the choice was made to assess sleepiness in terms of frequency rather than intensity and to propose explicit definitions and symptoms for sleepiness and severe sleepiness. The issue of self-rating will also apply to the timely use of countermeasures and to the evaluation of their effectiveness. However, it is possible to envisage future studies directly evaluating the efficiency of countermeasures, in an experimental environment such as a navigation simulator. This survey is a first attempt to identify the most relevant and widely used ones. It also makes it possible to target more precisely the contexts in which field studies on sleepiness among watch officers could be carried out.

The questionnaire answers may also be subject to social desirability bias. According to maritime legislation, the inability to stand watch for any reason (including fatigue) should result in a replacement and the officer must not navigate while drowsy. Similarly, some of the countermeasures proposed in this study are prohibited on the bridge while navigating (naps, breaks), which should encourage respondents to minimize these behaviors. In this study, a significant number of participants mentioned having these behaviors, as well as severe sleepiness and falling asleep, which suggests that social desirability bias was limited.

## 5. Conclusions

Merchant navy officers suffer from sleep restrictions which, in addition to affecting their health, pose a risk to maritime navigation by affecting alertness. The frequency of severe sleepiness and the risk of falling asleep during the watch are prevalent in the officer population. Sleepiness during maritime navigation is strongly linked to the possibilities of sleep on board and there is evidence that the opportunity to sleep varies as a function of organizations and workload rather than on the hours scheduled. The results indicate that officers frequently use caffeine, which is proven to be effective to counteract sleepiness, and also use alertness-enhancing activities that they consider to be effective although they are not validated. However, they are less inclined to be replaced in case of unfitness for duty, and very few have ever withdrawn from a shift due to severe sleepiness, while it is prevalent. These results indicate that strategies used for sleep management and sleepiness prevention during maritime navigation should be focused more toward sleep duration, safety culture, and the improvement of countermeasures to sleepiness.

## Figures and Tables

**Table 1:** Distribution of the responses for the variable “sleep requirements” for the duration of embarkation, the maritime sector, and the watch system.

Variables		Sufficient Sleep	Insufficient Sleep	Total
Duration of the trips at sea	Short <2 months	31	46	77
	Long >2 months	57	32	89
Maritime sector	Specialized ships	29	15	44
	Freight transport	24	17	41
	Passengers transport	19	36	55
Shift system	12 on-12 off 4 on-8 off 6 on-6 off	18 50 10	5 48 13	23 98 23

## Data Availability

Data will be shared upon specific request.
